# Stable Constitutive BiP Expression Prolongs Late-Stage GLP-1–Fc Accumulation in CHO Cells

**DOI:** 10.3390/bioengineering13091001

**Published:** 2026-08-28

**Authors:** Rolan R. Shaifutdinov, Fedor A. Krukov, Anastasia S. Samok, Ivan I. Vorobiev, Nadezhda A. Orlova

**Affiliations:** Laboratory of Mammalian Cell Bioengineering, Skryabin Institute of Bioengineering, Federal Research Centre ‘Fundamentals of Biotechnology’ of the Russian Academy of Sciences, 60 let Oktjabrja pr-t 7, bld. 1, 117312 Moscow, Russia; urtmyakowski@yandex.ru (R.R.S.); feodorkru05@gmail.com (F.A.K.); samok.nastya@mail.ru (A.S.S.); nobiol@gmail.com (N.A.O.)

**Keywords:** Chinese hamster ovary cells, CHO cell engineering, BiP, GRP78, HSPA5, endoplasmic reticulum stress, unfolded protein response, Fc-fusion protein, dulaglutide, fed-batch culture

## Abstract

Chinese hamster ovary (CHO) cells are the principal platform for manufacturing recombinant biopharmaceuticals, but sustained production can exceed the protein-folding capacity of the endoplasmic reticulum (ER). Binding immunoglobulin protein (BiP/GRP78), encoded by *Hspa5*, is a major ER chaperone that supports folding and modulates unfolded protein response (UPR) signaling, yet increasing BiP abundance has produced beneficial or inhibitory outcomes depending on the recombinant product. Here, we introduced an additional Chinese hamster Hspa5 coding sequence under the constitutive EEF1A1 promoter into a CHO line producing a dulaglutide analogue comprising a modified GLP-1 peptide fused to human IgG4 Fc and compared the resulting stable DUL-BiP population with an empty-vector DUL-Neo control. At constant 37 °C, the two populations showed similar viable cell density and viability trajectories, whereas DUL-BiP cells continued extracellular GLP-1–Fc accumulation for approximately two days after accumulation in DUL-Neo cells had slowed and reached a 40% higher final titer in this experiment. Two independent temperature-shift experiments showed the same direction of the final-titer difference, although the increases were smaller and were accompanied by reduced peak viable cell density. Semi-quantitative immunoblot analysis of samples from the constant-temperature experiment showed a comparatively stable total BiP signal in DUL-BiP cells and a pronounced late increase in DUL-Neo cells, together with temporal changes in cleaved ATF6, eIF2α phosphorylation, and CHOP in both populations. Intracellular GLP-1–Fc did not progressively accumulate. Because the immunoblot time courses were derived from one biological fed-batch experiment, they are interpreted descriptively rather than as independent quantitative biological replicates. Stable constitutive expression of host-derived BiP was therefore associated with prolonged late-stage GLP-1–Fc accumulation in the producer population examined. These kinetics are compatible with a model in which the timing of ER chaperone supply influences the maintenance of productive output, while the underlying mechanism and generality to other producer populations or recombinant products remain to be established.

## 1. Introduction

Chinese hamster ovary (CHO) cells can produce gram-per-liter quantities of complex therapeutic proteins, but their secretory capacity remains product-dependent. As expression increases, the limiting step often shifts from transgene output to the ability of the endoplasmic reticulum (ER) to fold, assemble, and traffic newly synthesized proteins. Slowly folding or aggregation-prone products can overload this system, particularly in late high-density fed-batch culture. Although antibody-producing CHO cells often upregulate ER folding factors such as binding immunoglobulin protein (BiP), no single molecular signature reliably identifies high producers across clones and product classes [1,2,3].

BiP has repeatedly been associated with the producer phenotype, but the direction of the association depends on the product. In one fusion-protein system, BiP abundance was 2.0- to 2.8-fold higher in the high-producing culture throughout the process and generally tracked intracellular product levels [4]. In our previous study of CHO clones producing B-domain-deleted factor VIII, the highest producer showed an approximately threefold increase in BiP mRNA, whereas the other clones remained near parental levels [5]. By contrast, Nissom et al. reported *Hspa5* downregulation in a high GFP-producing clone, with little change in other canonical UPR chaperones, consistent with more efficient protein processing and reduced ER stress [6]. BiP is therefore not a universal marker of productivity; rather, it reflects the balance between recombinant load and ER folding capacity.

BiP, also known as GRP78 and encoded by *HSPA5*/*Hspa5*, is the major Hsp70-family chaperone in the ER lumen. It binds exposed hydrophobic regions of nascent or misfolded proteins and, through an ATP-dependent cycle regulated by J-domain proteins and nucleotide exchange factors, promotes folding, prevents aggregation, supports assembly, and directs terminally misfolded proteins to ER-associated degradation. Because BiP also participates in UPR regulation, increasing its abundance may both expand folding capacity and raise the threshold for stress signaling. BiP-overexpressing CHO cells show weaker induction of endogenous GRP78, GRP94, and CHOP under stress, suggesting a delayed transition from adaptive to damaging ER stress [7,8,9,10,11].

These properties make BiP an attractive candidate for improving late-stage culture performance. Direct GRP78 overexpression reduced caspase-3 activation, protected CHO cells from serum deprivation and oxidative stress, and increased transient antibody accumulation [12]. Similarly, BiP inducer X (BIX) increased BiP and other ER chaperones, reduced CHOP and cleaved caspase-3, prolonged viability, and increased monoclonal antibody accumulation in chemically stressed CHO cultures [13]. Overexpression of KDELR1 also improved IgG-specific productivity, particularly during the deceleration phase, underscoring the importance of ER chaperone availability at late culture stages [14]. These findings suggest that increasing BiP may enhance final titer not only by supporting folding, but also by sustaining ER function and secretion during prolonged cultivation.

BiP can also act as a retention factor, so its overexpression is not universally beneficial. This limitation is particularly clear for factor VIII, where increased recombinant load induced GRP78 and GRP94 and caused pronounced ER dilation, yet secretion remained strongly bottlenecked post-translationally [15]. Constitutive GRP78 overexpression subsequently blocked secretion of mutant factor VIII and reduced von Willebrand factor secretion, an effect linked to direct substrate binding by GRP78 [7]. In IgG-producing cells, incomplete heavy chains bind BiP until light-chain-dependent assembly masks the retention sites; accordingly, BiP overexpression can reduce IgG-specific productivity when the light chain is limiting [16,17]. These observations indicate that BiP induction may report folding stress without necessarily identifying BiP itself as the limiting factor.

Product context is therefore critical, especially for Fc-fusion proteins. Unlike conventional antibodies, Fc-fusions do not require heavy-chain/light-chain pairing, although they still depend on ER translocation, disulfide-bond formation, glycosylation, and Fc dimerization. Their secretion is strongly influenced by the folding behavior of the fused partner domain, which can introduce new intermediates, protease-sensitive regions, and aggregation-prone surfaces. In IgG4 Fc-fusion producers, intracellular clipping and aggregation have been linked to changes in folding- and UPR-related proteins, whereas lower translation rates or reduced cultivation temperature improved product quality [18,19,20].

Dulaglutide is structurally simpler than many Fc-fusion therapeutics. Each chain contains a modified 31-residue GLP-1 peptide linked to the human IgG4 Fc region, and two identical chains form a covalent homodimer. This design avoids the heavy-chain/light-chain stoichiometric constraint of IgG while retaining the need for ER folding, disulfide-bond formation, N-glycosylation, and dimer assembly. These features make a constitutive BiP-supplementation strategy biologically distinct from the antibody systems in which increased BiP can reinforce retention of unassembled heavy chains [21].

Here, we investigated whether stable constitutive expression of the host-derived Chinese hamster Hspa5 coding sequence could alter the performance of a CHO cell line producing a dulaglutide analogue. The additional *Hspa5* cassette is driven by the Chinese hamster EEF1A1 promoter; throughout this study, the term stable constitutive BiP expression refers to this promoter-driven expression input and does not imply a fixed total intracellular BiP concentration. We hypothesized that providing an additional constitutive source of BiP could alter the balance between ER folding demand and chaperone supply during prolonged fed-batch cultivation and thereby help maintain productive output as the culture aged.

## 2. Materials and Methods

### 2.1. Cell Line

The clonal CHO 4BGD/Dul#73 cell line (DUL73) produces a dulaglutide analogue consisting of a GLP-1 peptide fused to the Fc region of human IgG4 [22]. The cell line was generated in-house from the CHO 4BGD host, which carries homozygous disruptions of the pro-apoptotic genes *Bak1* and *Bax* and the selection-marker genes *Dhfr* and *Glul*, together with genome-integrated expression cassettes containing additional copies of *Bcl-2* and *Beclin-1* [23]. The DUL73 clone contains two independently selected *EEF1A1*-based expression cassettes encoding the same GLP-1–Fc product and linked to DHFR- and GS-based selection systems. These constructs were derived from the Chinese hamster *EEF1A1* promoter and Epstein–Barr virus terminal-repeat vector platform developed previously for stable high-level expression in CHO cells [24].

### 2.2. Construction of the BiP Expression Vector and Generation of Engineered Cell Lines

#### 2.2.1. Amplification and Cloning of the Chinese Hamster Hspa5 Coding Sequence

The reference sequences used for comparison were *Cricetulus griseus Hspa5* mRNA NM_001246739.2 and protein NP_001233668.1, and *Homo sapiens HSPA5* mRNA NM_005347.5 and protein NP_005338.1. Total RNA was isolated from 2 × 10^6^ CHO 4BGD cells using the RNeasy Mini Kit (Qiagen, Hilden, Germany) according to the manufacturer’s protocol. RNA concentration was determined by UV spectrophotometry, and purity was assessed from the A_260_/A_280_ ratio, which was within the range of 1.8–2.0. First-strand cDNA was synthesized from 2 µL of the RNA preparation using the BioMaster RNAscribe RT Plus (5×) Kit (R02-100; Biolabmix, Novosibirsk, Russia). Reverse transcription was performed according to the protocol recommended for long templates, including a 60 min incubation at 55 °C.

The open reading frame of Chinese hamster *Hspa5*, encoding BiP/GRP78, was amplified from the resulting cDNA using the Encyclo Plus PCR Kit (Evrogen, Moscow, Russia). Oligonucleotides were synthesized by Lumiprobe (Westminster, MD, USA). Semi-nested amplification was performed in a single tube with two forward primers and one common reverse primer:

AD-Grp78AbsF, 5′-ACCTCGAGGCCGCCACCATGAAGTTCCCTATGGTGGCG-3′;

GRP78-inF, 5′-TATGGTGGCGGCGGCGCTGCT-3′;

AD-Grp78NheR, 5′-TGCTAGCCTACAACTCATCTTTTTCTGATGTATCC-3′.

The reaction contained both forward primers and the common reverse primer. PCR cycling comprised an initial denaturation at 95 °C for 2 min, followed by 38 cycles of 95 °C for 15 s, 57 °C for 15 s, and 72 °C for 90 s, with a final extension at 72 °C for 5 min.

The PCR product was analyzed by electrophoresis in a 1% agarose gel prepared in 1× TBE buffer and purified using the Cleanup Standard DNA purification kit (Evrogen). DNA concentration was measured with a Qubit Fluorometer and the dsDNA HS Assay Kit (Thermo Fisher Scientific Inc., Waltham, MA, USA). An in-house plasmid DNA preparation quantified by UV spectrophotometry was used as an external concentration control.

The purified product was inserted into the pCE3 cloning vector using the 5 min TOPO-Blunt Cloning Kit (C602-02; Vazyme, Nanjing, China). The cloning mixture was introduced into chemically competent *Escherichia coli* TOP10 cells (Invitrogen, Carlsbad, CA, USA), prepared in-house by the Inoue method. Transformants were selected on 2 × YT agar containing ampicillin at 50 µg/mL. Candidate colonies were screened by PCR using ScreenMix HS (Evrogen), and plasmid DNA was isolated using the Plasmid-250-mini Kit (Biolabmix, Novosibirsk, Russia). The resulting plasmid was designated pCE3-BiP. The complete insert was verified by Sanger sequencing at the Interinstitutional Center for Collective Use “GENOME,” Engelhardt Institute of Molecular Biology, Russian Academy of Sciences, using standard M13 forward and reverse primers and the following *Hspa5*-specific primers: RT-BIP-F, 5′-ACTGCTTGATGTATGTCCTCTTAC-3′; RT-BIP-R, 5′-TGTTAGGGGTCGTTCACCTTC-3′.

#### 2.2.2. Assembly of the p1.2-Neo-BiP Expression Plasmid

The p1.2-Neo vector belongs to the family of Chinese hamster *EEF1A1*-based plasmids developed for stable monocistronic expression in CHO cells. The target-gene expression unit contains the Chinese hamster *EEF1A1* upstream regulatory region and promoter, the first intron, a cloning region, and downstream transcription-termination and polyadenylation sequences. The vector also contains an Epstein–Barr virus terminal-repeat concatemer (EBVTR), which supports the establishment of stable transfected CHO populations [25]. Stable transfectants are selected through a separate neomycin phosphotransferase expression cassette controlled by the SV40 promoter and SV40 termination and polyadenylation sequences. This marker allowed the BiP expression cassette to be introduced independently of the DHFR/MTX- and GS/MSX-based selection systems already present in the producer.

The verified Chinese hamster *Hspa5* insert was excised from pCE3-BiP with AbsI and AsuNHI restriction endonucleases (Sibenzyme, Novosibirsk, Russia), separated on a 1% agarose gel, and purified using the Cleanup Standard DNA purification kit (Evrogen). The p1.2-Neo vector was linearized with the same enzymes and purified by ethanol precipitation. Ligation was performed with T4 ligase (Sibenzyme). The ligation mixture was transformed into chemically competent *E. coli* TOP10 cells, and transformants were selected on 2 × YT agar containing ampicillin at 50 µg/mL. Candidate clones were screened by colony PCR and restriction analysis. Plasmid DNA for clone screening was isolated using the Plasmid-250-mini Kit (Biolabmix).

The resulting construct, designated p1.2-Neo-BiP, contained the Chinese hamster *Hspa5* coding sequence under the control of the *EEF1A1* promoter–intron–terminator unit. The cloned sequence retained the N-terminal signal peptide required for ER translocation and the C-terminal KDEL retention motif. Correct plasmid assembly and integrity of the *Hspa5* coding sequence were confirmed by complete Oxford Nanopore sequencing performed by Cloning Facility JSC (Moscow, Russia). Because the cassette uses the constitutive EEF1A1 regulatory unit, we refer to the engineered condition as stable constitutive BiP expression; this terminology describes the expression design rather than a claim that total cellular BiP abundance is constant.

Transfection-grade p1.2-Neo-BiP plasmid DNA was prepared from bacterial cultures using the Plasmid-20 maxi Kit (Biolabmix), followed by additional ethanol precipitation. The corresponding empty p1.2-Neo plasmid was prepared in the same manner and used to generate matched vector-control cells.

#### 2.2.3. Stable Transfection and Selection

Twenty-four hours before transfection, CHO 4BGD/Dul#73 cells were passaged by dilution with prewarmed culture medium. For each transfection, 1 × 10^7^ viable cells were collected by centrifugation and washed once with DPBS (PanEco, Moscow, Russia). The cell pellet was resuspended in 100 µL of SBB buffer containing 250 mM sucrose, 1 mM MgCl_2_, and DPBS. The cell suspension was supplemented with 50 µg of either p1.2-Neo-BiP or the corresponding empty p1.2-Neo control plasmid. Plasmid DNA was prepared in SBB buffer at a concentration of 1–3 mg/mL.

To assess transfection efficiency, 2.5 µg of the pEGFP-N2 reporter plasmid was added to each transfection mixture. Electroporation was performed using the Neon Transfection System and Neon 100 µL Transfection Kit (Invitrogen, Carlsbad, CA, USA) with a single 20 ms pulse at 1700 V.

Immediately after electroporation, cells were transferred to 30 mL of prewarmed custom-manufactured EmCD CHO 101 medium lacking hypoxanthine and thymidine (Eminence, Suzhou, China). No exogenous L-glutamine was added. Cells were allowed to recover for 48 h without selection pressure. G418 and methotrexate were then added to final concentrations of 0.5 mg/mL and 200 nM, respectively. Selection was continued until viability recovered above 90% and stable proliferation resumed.

The polyclonal populations transfected with p1.2-Neo-BiP and the empty p1.2-Neo vector were designated DUL-BiP and DUL-Neo, respectively. The selected populations were expanded without cell cloning and used for fed-batch cultivation. Thus, the study compared one independently selected DUL-BiP polyclonal population with one matched DUL-Neo polyclonal population, both derived from the same DUL73 producer clone. Following primary selection, both populations were routinely maintained in hypoxanthine- and thymidine-free EmCD CHO 101 medium without added L-glutamine and supplemented with 100 µg/mL G418 and 200 nM methotrexate.

### 2.3. Cell Cultivation and Fed-Batch Experiments

#### 2.3.1. Routine Expansion and Batch Culture

DUL-Neo, DUL-BiP and control CHO 4BGD cells were routinely maintained in 20 mL of custom-manufactured EmCD CHO 101 basal medium lacking hypoxanthine and thymidine (Eminence, Suzhou, China) in 125 mL vented Erlenmeyer flasks. Cultures were incubated at 37 °C in a humidified atmosphere containing 5% CO_2_ with orbital shaking at 140 rpm on a 10 mm orbit. For routine passaging, cells were inoculated at a viable cell density of 0.3–0.5 × 10^6^ cells/mL. The maintenance medium was supplemented with 200 nM methotrexate and 100 µg/mL G418 and no exogenous L-glutamine was added to DUL-Neo and DUL-BiP cultures. Parental CHO 4BGD cells were supplemented with HT and 4 mM glutamine instead of G418 and methotrexate.

#### 2.3.2. Fed-Batch Cultivation

Before fed-batch cultivation, DUL-Neo and DUL-BiP cells were passaged once in EmCD CHO CM110 prototype basal medium (Eminence, Suzhou, China) without methotrexate or G418 to allow adaptation to the experimental medium under selection-free conditions. Fed-batch cultures were established in 125 mL Erlenmeyer flasks with an initial working volume of 30 mL. Cells in the logarithmic growth phase were inoculated at a viable cell density of 1.0 × 10^6^ cells/mL. Neither methotrexate nor G418 was added during fed-batch cultivation. Flasks were incubated at 37 °C in a humidified atmosphere containing 5% CO_2_ with orbital shaking at 140 rpm on a 10 mm orbit.

The first culture samples were collected on day 3 immediately before feeding. The 110A and 110B prototype feeds (Eminence) were subsequently added every 24 h at volumes corresponding to 3% and 0.3% of the initial 30 mL culture volume, respectively; thus, each flask received 0.9 mL of 110A feed and 0.09 mL of 110B feed per feeding. Glucose concentration was monitored daily using an On Call Plus glucometer and the corresponding test strips (Acon, San Diego, CA, USA). When required, sterile 2 M glucose solution was added to raise the glucose concentration by 20 mM. For temperature-shift experiments, cultures were established and fed as described above. The incubation temperature was reduced from 37 °C to 34 °C on day 3 immediately after sampling and feeding and was maintained at 34 °C until the end of cultivation. Two independent fed-batch experiments were performed.

A 500 µL sample of culture suspension was collected daily from each flask. A 30 µL aliquot was used to determine viable cell density and viability by trypan blue exclusion. The remaining sample was centrifuged to separate the cells from the culture supernatant. Cell pellets and clarified supernatants were collected as duplicate technical aliquots. Cell pellets containing approximately 1.25–5 × 10^6^ cells were collected daily for protein analysis; when the routine sample did not provide sufficient material, an additional aliquot was collected. The cell pellets were washed once with phosphate-buffered saline. Cell pellets and supernatant aliquots were stored separately at −70 °C until analysis.

GLP-1–Fc concentration was determined in culture supernatants from day 3 onward. Equal amounts of total cellular protein were subsequently used for Western blot analysis, as described below. Cultivation was continued for 16 days, by which time viability had declined to approximately 80%. IVCD was calculated as the cumulative sum of the mean viable cell density over each consecutive cultivation interval multiplied by the duration of that interval.

### 2.4. Quantification of GLP-1–Fc by Analytical Protein A Chromatography

Culture samples were clarified by centrifugation at 2500 rpm for 5 min. The recovered supernatants were subjected to a second centrifugation at 13,500 rpm for 5 min to remove residual cells and particulate material. Clarified samples were transferred into polypropylene microinserts with conical bottoms (SN2004, 5.8 × 31.5 mm; ALWSCI Technologies, Shaoxing, China) placed in 1.1 mL screw-thread glass vials (1.1-STVG).

GLP-1–Fc concentration was determined by affinity chromatography using a BioCore Protein A analytical column (NanoChrom Technologies, Suzhou, China) connected to a Waters Alliance 2795 separation module equipped with a Waters 2996 photodiode array detector. Chromatographic data were acquired and processed using Empower 3 software (Waters, Milford, MA, USA). Mobile phase A consisted of 100 mM sodium phosphate buffer, pH 7.0, prepared from 39 mM sodium dihydrogen phosphate and 61 mM disodium hydrogen phosphate. Mobile phase B consisted of 100 mM sodium dihydrogen phosphate adjusted to pH 2.5 with phosphoric acid.

Chromatography was performed at a flow rate of 2.0 mL/min using the following program: 0.00–0.50 min, 0% mobile phase B; 0.50–0.51 min, a linear increase from 0% to 100% B; 0.51–1.50 min, 100% B; 1.50–1.51 min, a return from 100% to 0% B; and 1.51–2.50 min, 0% B for column re-equilibration. Protein elution was monitored by absorbance at 280 nm. All culture-supernatant and reference samples were injected at a volume of 50 µL.

Commercial Trulicity drug product was used as the dulaglutide reference preparation. Two reference loads containing 25 and 50 µg of dulaglutide were analyzed using the same chromatographic method. The GLP-1–Fc concentration in each culture sample was calculated independently from the integrated Protein A elution-peak area obtained for each reference load, taking sample dilution into account; the two resulting estimates were then averaged.

Changes in extracellular GLP-1–Fc concentration over time were used to assess continued product accumulation during fed-batch cultivation. Apparent cell-specific productivity (q_P_) was calculated for each interval between two consecutive GLP-1-Fc titer measurements. Because the volume of added feeds was approximately balanced by sampling and evaporation, changes in culture volume were neglected. With GLP-1–Fc titer expressed in mg/L and viable cell density in 10^6^ cells/mL, q_P_ was calculated as shown in Equation (1):

(1) $$q_{P}=\frac{C_{2}-C_{1}}{\left(\frac{{VCD}_{1}+{VCD}_{2}}{2}\right)\left(t_{2}-t_{1}\right)}$$

where C_1_ and C_2_ are GLP-1–Fc titers (mg/L) at the beginning and end of the interval, VCD_1_ and VCD_2_ are viable cell densities (10^6^ cells/mL), and t_1_ and t_2_ are the corresponding culture times (days). With these units, q_P_ is expressed directly in pg/cell/day. Each q_P_ value was assigned to the midpoint of the corresponding interval. Negative q_P_ values arising from a decrease in the measured titer between consecutive sampling points were set to zero before smoothing. The corrected q_P_ values were smoothed separately for each biological repeat using a centered three-point moving average (Equation (2)):

(2) $$q_{P,i,smooth}=\frac{q_{P,i-1,corrected}+q_{P,i,corrected}+q_{P,i+1,corrected}}{3}$$

At the boundaries of each series, the moving average was calculated from the two available adjacent values. Biological repeats were processed and plotted separately and were not averaged.

### 2.5. Western Blot Analysis

Frozen cell pellets were resuspended in an ice-cold RIPA-based lysis solution composed of 50 mM Tris-HCl, pH 8.0, 150 mM NaCl, 1% Triton X-100, 0.1% sodium deoxycholate, 0.1% SDS, 1 mM EDTA, and 0.01% NaN_3_. Before lysis, 1 mM phenylmethylsulfonyl fluoride (Sigma-Aldrich, St. Louis, MO, USA), Antiase protease inhibitor mixture, and Antiphos AC/AL/S/T/Y phosphatase inhibitor mixture (BioInnLabs, Rostov-on-Don, Russia) were added to the buffer. Samples were incubated on ice for 30 min and then clarified by centrifugation at 13,400 rpm for 10 min.

Protein concentration in the soluble lysate fraction was measured using the Micro BCA assay (Sigma-Aldrich), with a 2 mg/mL bovine serum albumin solution used to prepare the calibration curve. Equal amounts of total protein were combined with reducing sample buffer containing 50 mM dithiothreitol. Unless specified otherwise, 5 µg of protein was applied to each lane of a 12% polyacrylamide gel.

Following electrophoretic separation, proteins were electrotransferred onto BioTrace NT nitrocellulose membranes (Pall Corporation, Port Washington, NY, USA) in a semi-dry apparatus. Transfer was performed for 2 h at a current density of 1 mA/cm^2^ using Towbin buffer supplemented with 20% ethanol and lacking SDS.

Nonspecific binding sites were saturated for 1 h at room temperature with 5% skimmed milk prepared in Tris-buffered saline containing 0.1% Tween 20. When several proteins of different molecular masses were analyzed on the same membrane, the membrane was cut into the appropriate molecular-weight regions before antibody incubation. Primary antibodies were applied overnight at 4 °C in blocking solution.

The following antibodies were used: rabbit anti-ATF6 (DF6009, Affinity Biosciences, Wuhan, China; 1:1000), rabbit anti-phospho-eIF2α (Ser51) (ARM2964, AssayVector Biotechnology Co., Ltd., Katy, TX, USA; 1:1000), rabbit anti-eIF2α (E-AB-62069, Elabscience Bionovation Inc., Houston, TX, USA; 1:1000), rabbit anti-DDIT3/CHOP (PAJ282Hu01, Cloud-Clone Corp., Wuhan, China; 1:1000), mouse anti-HSPA5/BiP (MAC343Mu21, Cloud-Clone,; 1:1000), mouse anti-calnexin (MAA280Hu22, Cloud-Clone; 1:1000), and mouse anti-GAPDH clone 6C5 (HyTest, Moscow, Russia; 1:10,000).

After primary-antibody incubation, membranes were rinsed three times with TBST (once for 15 min and twice for 5 min). Species-appropriate horseradish peroxidase-conjugated secondary antibodies were then applied for 1 h at room temperature. Goat anti-rabbit IgG(H + L)-HRP (E-AB-1194, Elabscience) and goat anti-mouse IgG-HRP (ab6789, Abcam, Cambridge, UK) were each used at a dilution of 1:2000.

Chemiluminescence was generated with Pierce ECL Western Blotting Substrate (Thermo Fisher Scientific, Waltham, MA, USA) and captured using a Fusion FX imaging system (Vilber Lourmat, Collégien, France). Band intensities were quantified in TotalLab 2009 software (Nonlinear Dynamics Ltd., Newcastle upon Tyne, UK). Unless otherwise specified below, antigen signals were normalized to the corresponding GAPDH signal from the same lane. Samples collected at successive cultivation time points were analyzed in independently processed immunoblots. Three independently processed immunoblots of the same biological sample series were quantified for BiP, calnexin and GAPDH, whereas two were quantified for the other antigens. These repeated immunoblots are technical processings of the same fed-batch sample series and are used to assess analytical reproducibility and temporal signal patterns, not biological variability.

For the daily BiP time-course analysis, DUL-Neo and DUL-BiP samples collected on days 3–16 were resolved on separate membranes. The membranes were blocked, incubated with antibodies, washed, developed, and imaged simultaneously under identical conditions. Two exposures providing quantifiable BiP signals were analyzed for each membrane. To align the intensity scales of the two exposures, values from one exposure were multiplied by a scaling coefficient calculated as the median ratio of corresponding band intensities between the exposures. The two exposure-derived values were then averaged for each time point. For each cell population, BiP values were normalized to the median signal measured on days 3–11. Because the two populations were resolved on separate membranes and normalized independently, these data are not used to estimate an absolute fold difference in BiP abundance between DUL-Neo and DUL-BiP cells.

For the Western blot analysis of all proteins for even culture days, samples collected on even cultivation days were resolved with DUL-Neo and DUL-BiP samples placed side by side on the same membrane. The intensity of each antigen band was divided by the corresponding GAPDH signal from the same lane. For each independently processed membrane, normalized values for both cell populations were expressed relative to the DUL-Neo day 4 sample analyzed on that membrane. CHOP values were expressed relative to the corresponding DUL-Neo day 6 sample because of the exceptionally strong DUL-Neo day 4 signal. The phospho-eIF2α/total eIF2α ratio was calculated from the corresponding GAPDH-normalized values and expressed relative to the DUL-Neo day 4 ratio. Individual technical trajectories and their arithmetic means were plotted. Three independently processed immunoblots were analyzed for BiP and calnexin, and two were analyzed for the other antigens. All samples were obtained from the constant-temperature fed-batch experiment. Repeated membrane processing represents technical measurement of the same biological sample series and is interpreted descriptively.

### 2.6. Data Presentation and Replication

The two temperature-shift fed-batch experiments were independent biological experiments and are plotted separately. The constant-temperature fed-batch experiment was performed once and is presented as one biological experiment. Technical replicate measurements, where available, are shown to document analytical variability and are not treated as independent biological observations. The immunoblot time courses were generated from samples collected during the single constant-temperature fed-batch experiment; repeated exposures or independently processed immunoblots therefore constitute technical measurements of the same biological sample series. No inferential hypothesis testing was applied to the single constant-temperature experiment or to the immunoblot time courses. Densitometric values and apparent qP trajectories are used descriptively to characterize temporal patterns.

## 3. Results

### 3.1. Generation and Initial Screening of GLP-1–Fc-Producing CHO Cells with Stable Constitutive BiP Expression

**Figure 1:**
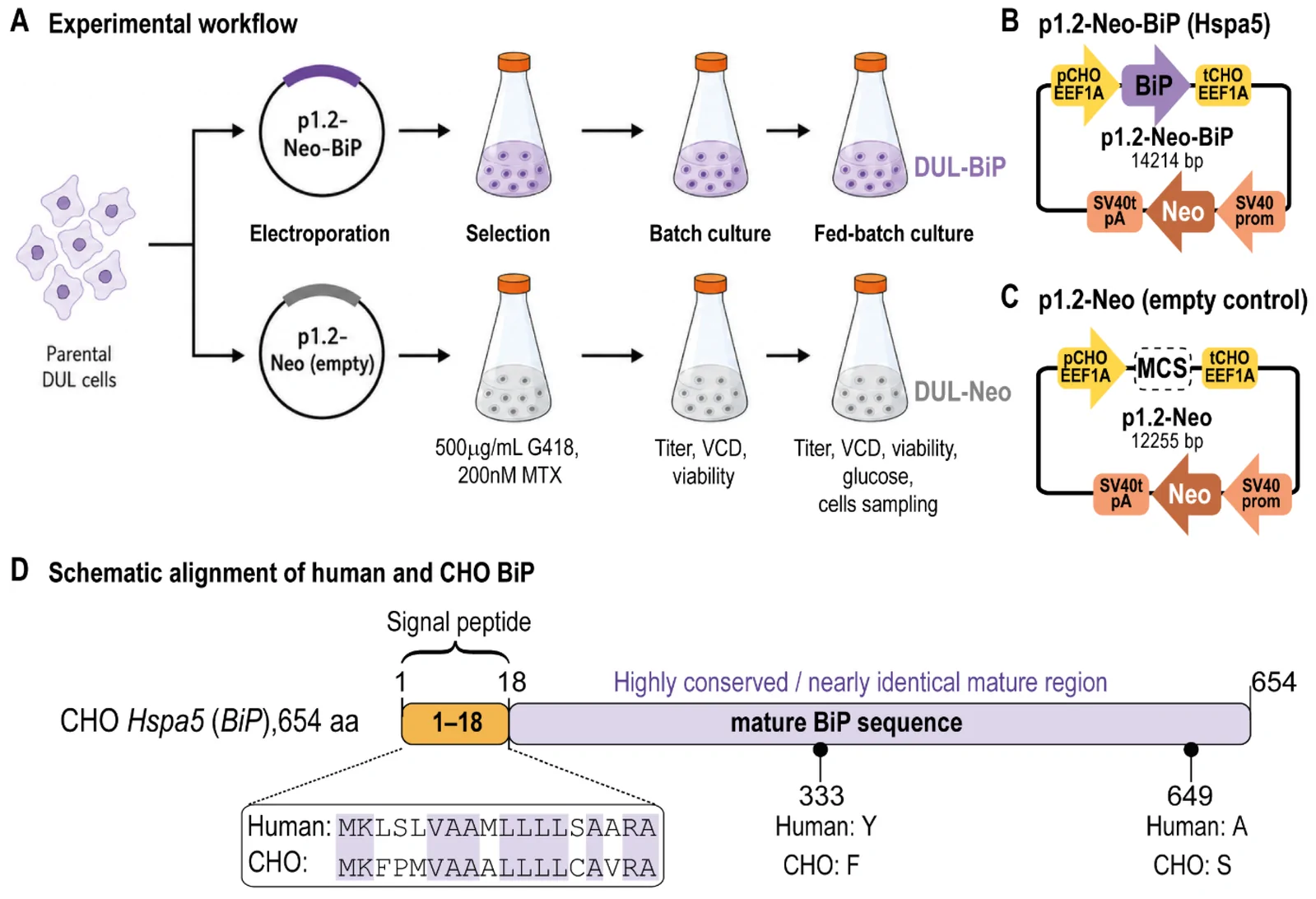
Generation of GLP-1–Fc-producing CHO cells with stable constitutive BiP expression. (**A**) Experimental workflow. Parental DUL73 cells were electroporated with p1.2-Neo-BiP or empty p1.2-Neo, selected with 500 µg/mL G418 and 200 nM MTX, screened in batch culture, and compared in fed-batch culture. (**B**) p1.2-Neo-BiP (*Hspa5*) plasmid. (**C**) Empty p1.2-Neo control plasmid. (**D**) Human and CHO BiP sequence comparison. The signal peptides and the two substitutions in the otherwise nearly identical mature proteins are shown; shading indicates identical residues. pCHO *EEF1A1*, tCHO *EEF1A1*, promoter/intron and terminator elements derived from Chinese hamster *Eef1a1*; Neo, neomycin phosphotransferase conferring G418 resistance; SV40 prom, SV40 promoter; SV40t pA, SV40 polyadenylation signal; MCS, multiple cloning site; MTX, methotrexate; VCD, viable cell density. Plasmid maps are not drawn to scale.

To determine whether an additional constitutive *Hspa5* expression input could alter production of the dimeric GLP-1–Fc fusion protein, DUL73 cells were stably transfected in parallel with the p1.2-Neo-BiP expression plasmid or the corresponding empty p1.2-Neo vector (Figure 1A). Stable G418-resistant polyclonal populations were designated DUL-BiP and DUL-Neo, respectively.

**Figure 2:**
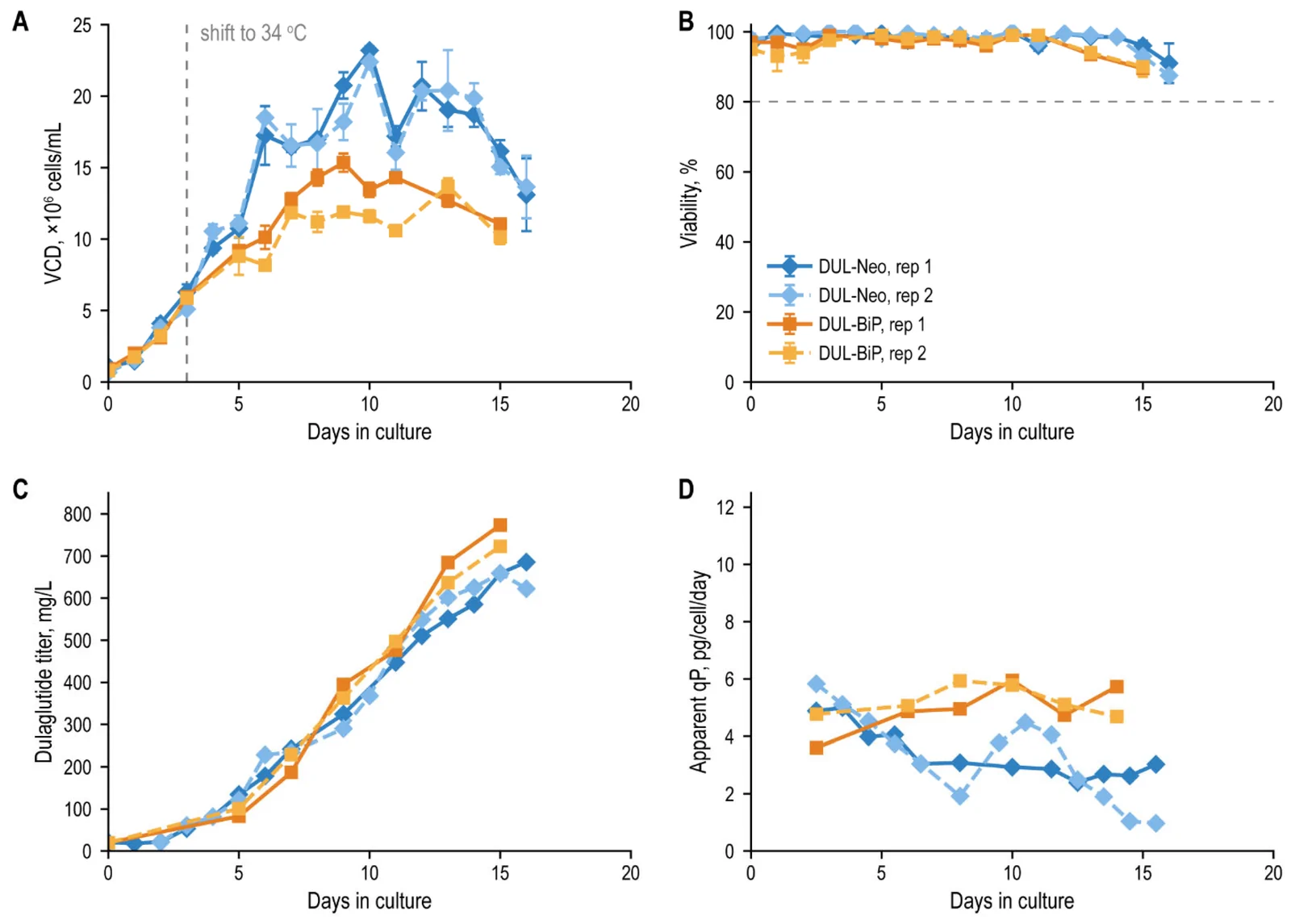
Growth, viability, GLP-1–Fc production, and apparent cell-specific productivity of DUL-Neo and DUL-BiP populations during fed-batch cultivation with a temperature shift to 34 °C. (**A**) Viable cell density (VCD); the vertical dashed line indicates the temperature shift from 37 to 34 °C on day 3. (**B**) Cell viability; the horizontal dashed line indicates 80% viability. (**C**) GLP-1–Fc titer. (**D**) Smoothed apparent cell-specific productivity (q_P_). Two biological repeats are shown separately. DUL-Neo controls are shown in blue and DUL-BiP cells in orange; different shades and line styles distinguish biological repeats. Error bars indicate the SD of two technical measurements where available.

The p1.2-Neo-BiP construct expressed the Chinese hamster *Hspa5* coding sequence as a monocistronic transcript under the control of the Chinese hamster *EEF1A1* promoter–intron–terminator unit (Figure 1B), and it retained the native BiP signal peptide and C-terminal KDEL ER-retention motif coding sequences (Figure 1D). The empty-vector population (Figure 1C) was generated using the same transfection and selection procedure, providing a matched control for the effects of stable transfection, G418 selection, and random integration of the p1.2-Neo backbone. Preliminary batch-culture screening did not reveal an evident difference in growth or product accumulation. The populations were therefore evaluated in fed-batch culture with a temperature shift to 34 °C on day 3, as described previously [26]. A modest final-titer difference in the same direction was observed in two independent temperature-shift experiments (723–773 vs. 622–686 mg/L for DUL-BiP and DUL-Neo, respectively), together with lower peak cell density in DUL-BiP cultures (approximately 15 × 10^6^ vs. 23 × 10^6^ cells/mL; Figure 2A,C). Both populations had a similar cultivation time before viability declined below 80% (Figure 2B). Further fed-batch testing was performed at constant 37 °C to assess the phenotype without a temperature shift.

### 3.2. DUL-BiP Cells Show Prolonged GLP-1–Fc Accumulation During Constant-Temperature Fed-Batch Culture

**Figure 3:**
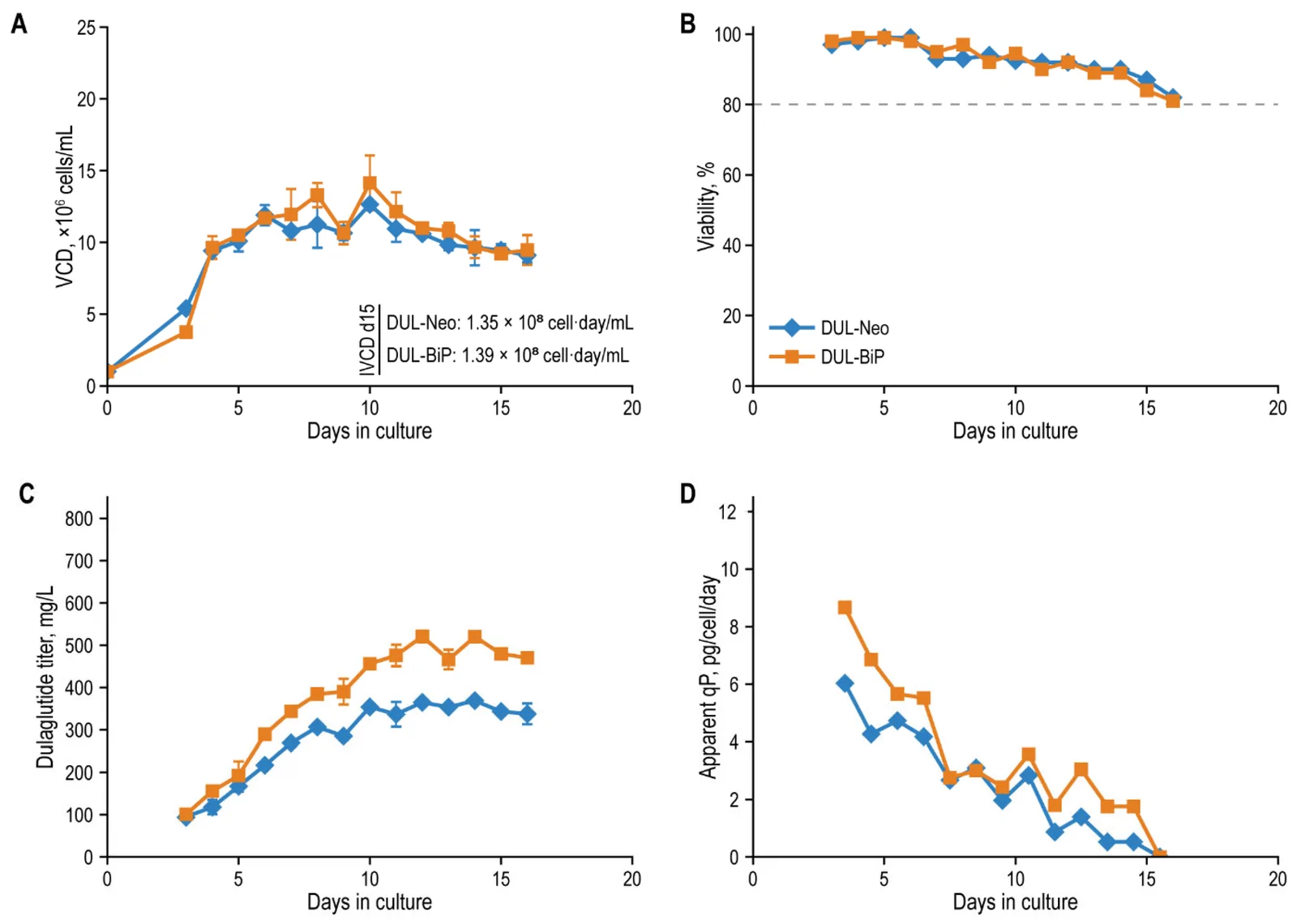
Growth, viability, GLP-1–Fc production, and apparent cell-specific productivity of DUL-Neo and DUL-BiP populations during fed-batch cultivation at constant 37 °C. (**A**) Viable cell density (VCD). (**B**) Cell viability; the horizontal dashed line indicates 80% viability. (**C**) GLP-1–Fc titer. (**D**) Smoothed apparent cell-specific productivity (q_P_). One biological fed-batch experiment is shown. DUL-Neo controls are shown in blue and DUL-BiP cells in orange. Error bars, where present, indicate variation between technical measurements and do not represent biological replication.

Fed-batch cultivation at constant 37 °C with the same feeding scheme produced similar cell-growth curves and viability dynamics for the DUL-BiP and DUL-Neo populations (Figure 3A,B). Thus, the higher product accumulation observed in DUL-BiP was not accompanied by an evident growth or viability advantage in this experiment. The titer curves were not completely superimposable from the early sampled phase, and we therefore do not interpret the phenotype as exclusively late-onset. The clearest kinetic distinction emerged after approximately day 11, when extracellular GLP-1–Fc accumulation in DUL-Neo slowed while DUL-BiP cultures continued to accumulate product for approximately two additional days (Figure 3C,D). The final GLP-1–Fc titer in this biological experiment was 511 mg/L for DUL-BiP and 365 mg/L for DUL-Neo, corresponding to an approximately 40% difference.

Because the two populations showed similar viable-cell-density profiles, the final-titer difference could not be explained by increased biomass. The production kinetics instead indicate a longer period of net extracellular GLP-1–Fc accumulation in DUL-BiP during the late phase of this constant-temperature fed-batch experiment. Apparent qP is presented as a descriptive kinetic measure rather than as an independently replicated biological endpoint.

### 3.3. Stable Constitutive BiP Expression Is Associated with a Distinct Temporal Pattern of Total BiP Abundance

**Figure 4:**
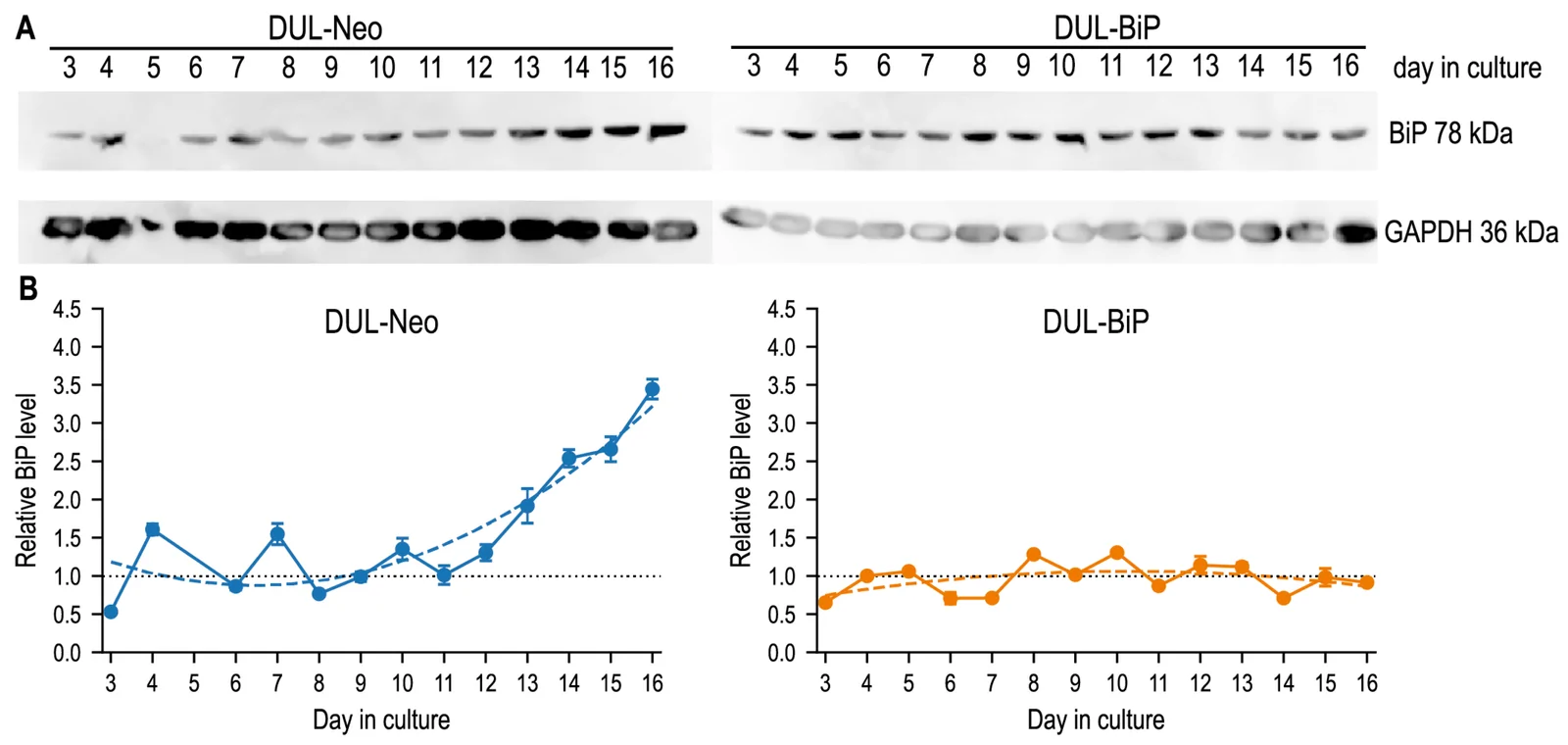
Temporal pattern of total BiP abundance in DUL-Neo and DUL-BiP cells during fed-batch cultivation at constant temperature. (**A**) Immunoblotting of BiP and GAPDH using two membranes processed and imaged simultaneously under identical conditions. (**B**) Densitometric BiP values normalized to the median value obtained on days 3–11 for the corresponding cell population. Values are derived from two exposures of the same membrane and represent technical measurements of one biological sample series. Because DUL-Neo and DUL-BiP were resolved on separate membranes and normalized independently, the analysis is intended to show within-population temporal patterns rather than absolute differences in BiP abundance between populations. No inferential statistical test was applied.

To examine how total BiP abundance changed during prolonged cultivation, intracellular proteins were analyzed at successive fed-batch time points by Western blotting (Figure 4A). From day 3 to approximately day 12, the BiP signal within each population was relatively stable. During later culture, DUL-Neo showed a pronounced increase in total BiP, with the strongest signal near day 16, whereas DUL-BiP did not show a comparable late increase (Figure 4B). In DUL-Neo, this late rise occurred around the period in which extracellular product accumulation was slowing. Because DUL-Neo and DUL-BiP samples in Figure 4 were resolved on separate membranes and normalized independently within each population, these data characterize temporal trajectories and do not support an absolute fold comparison of total BiP abundance between the two populations.

### 3.4. Increased Extracellular Production Is Not Accompanied by Progressive Intracellular Accumulation of GLP-1–Fc

**Figure 5:**
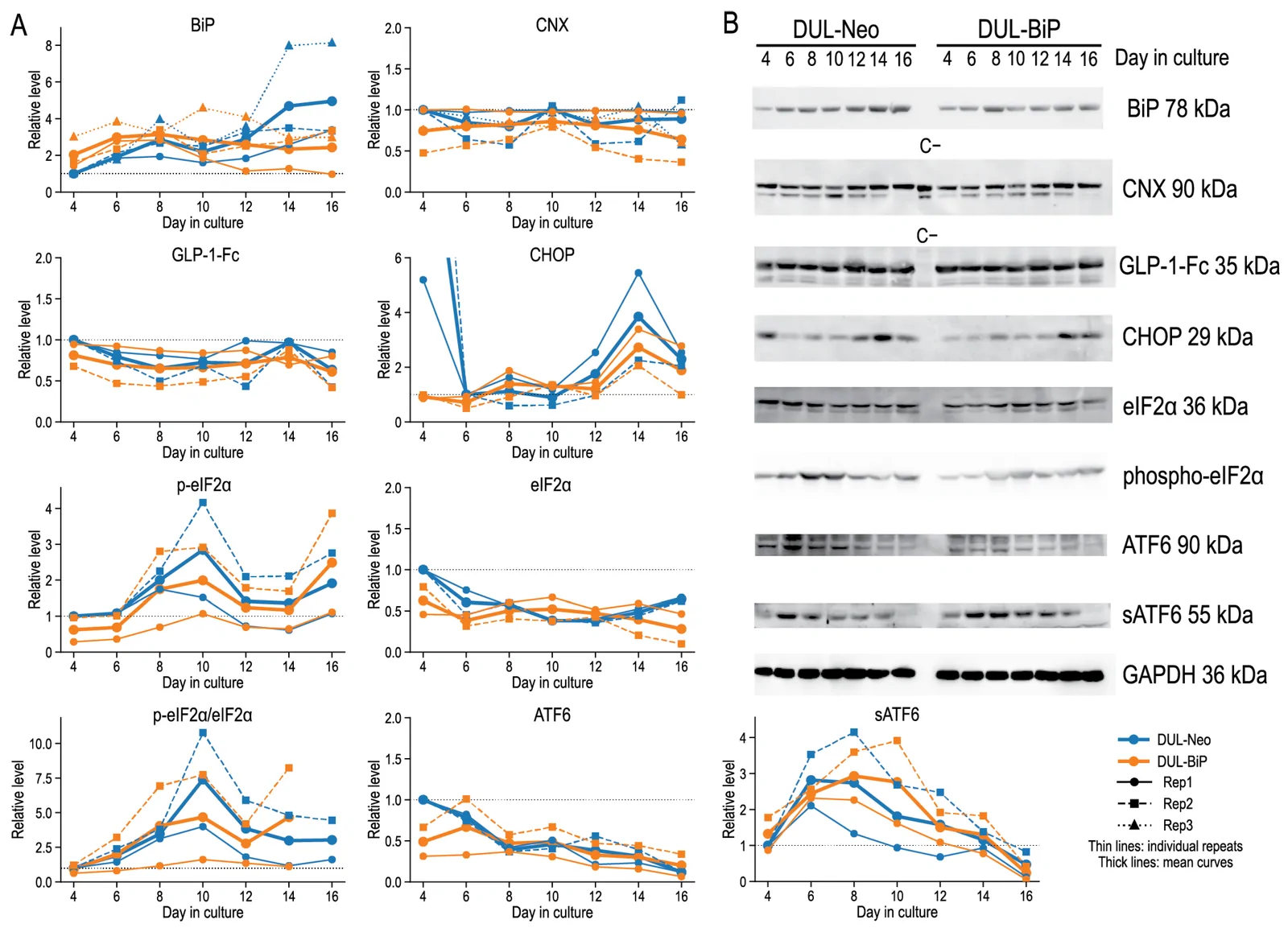
Temporal dynamics of BiP, intracellular GLP-1–Fc, and UPR-associated proteins during fed-batch cultivation. (**A**) Representative Western blots for BiP, calnexin, intracellular GLP-1–Fc, CHOP, total eIF2α, phosphorylated eIF2α, full-length ATF6, cleaved ATF6, and GAPDH. (**B**) Densitometric analysis of the indicated proteins and the phospho-eIF2α/total eIF2α ratio during cultivation. Antigen signals were first normalized to the corresponding GAPDH signal from the same lane. For each membrane, values for both populations were then expressed relative to the DUL-Neo day 4 sample analyzed on that membrane; CHOP values were expressed relative to the corresponding DUL-Neo day 6 sample. The DUL-Neo day 4 CHOP value exceeded the displayed *y*-axis range. GAPDH and BiP were probed together on the same membrane; other antigens were hybridized individually using membrane halves. C−, parental CHO 4BGD sample included as a GLP-1–Fc-negative control on the CNX/GLP-1–Fc membrane; the corresponding positions on the other membranes were left empty. Individual technical trajectories and arithmetic means are shown; three independently processed immunoblots of the same biological sample series were analyzed for BiP and calnexin and two for the other antigens. All samples were obtained from the single constant-temperature fed-batch experiment shown in Figure 3. The densitometry is descriptive; no inferential statistical test was applied.

The temporal profiles of BiP and other intracellular proteins were examined in additional Western blot series using samples collected on even cultivation days from day 4 to day 16 (Figure 5A). In each independently processed blot, DUL-Neo and DUL-BiP samples were placed side by side on the same membrane, reducing within-blot membrane effects. For each membrane, antigen signals were normalized to the corresponding GAPDH signal. Values for both populations were then expressed relative to the DUL-Neo day 4 sample analyzed on the same membrane, except for CHOP, which was normalized to DUL-Neo day 6. Because all of these blots used the same biological fed-batch sample series, the densitometric trajectories are interpreted semi-quantitatively and descriptively.

The side-by-side blots supported the same principal temporal distinction seen in Figure 4: total BiP in DUL-BiP showed a comparatively stable trajectory across cultivation, whereas DUL-Neo developed a pronounced increase during the late phase (Figure 5B). Mean normalized signals differed at some individual time points, but technical reprocessing of a single biological sample series does not provide an independent estimate of a biological fold change between the populations. We therefore do not assign a specific fold increase in BiP to DUL-BiP cells.

Calnexin abundance showed no distinctive temporal changes (Figure 5B, CNX). The late increase in BiP in DUL-Neo cells was therefore not accompanied by a comparable increase in calnexin.

Intracellular GLP-1–Fc did not show progressive accumulation in either population (Figure 5B, GLP-1-Fc). Thus, the higher extracellular GLP-1–Fc accumulation in DUL-BiP was not accompanied by a progressively expanding steady-state intracellular product pool. This observation does not by itself distinguish altered secretion, folding, processing, or intracellular degradation.

### 3.5. Fed-Batch Cultivation Produces Temporally Distinct Changes in CHOP, eIF2α Phosphorylation, and ATF6 Forms

CHOP abundance was evaluated relative to the DUL-Neo day 6 sample because the day 4 DUL-Neo sample showed a markedly different early signal profile (Figure 5B, CHOP). From day 6, CHOP remained comparatively low through days 8–12 and increased during the late phase in both populations. The technical trajectories suggested a stronger late signal in DUL-Neo, but this difference is interpreted descriptively because the samples derive from one biological fed-batch experiment.

Phosphorylated eIF2α showed two periods of increased signal in both cultures, with an initial increase on days 10–12 and a second rise on the culture-termination day (Figure 5B, p-eIF2α and p-eIF2α/eIF2α). Total eIF2α was generally more abundant in the DUL-Neo technical series and increased again during the late phase in that culture. Consequently, the p-eIF2α/eIF2α ratio showed a maximum around day 10 in DUL-Neo, whereas DUL-BiP showed increases around days 10 and 16. The day 16 ratio for DUL-BiP is out of scale because the total eIF2α signal was too weak for reliable quantification, corresponding values are presented in Supplementary Materials. These trajectories are descriptive and are not interpreted as statistically replicated differences between populations.

Full-length ATF6, detected as a series of bands around 90 kDa, decreased progressively in both cell populations (Figure 5B, ATF6). In DUL-Neo, the ATF6 signal declined after day 4 and was barely detectable by day 16 at the sample load used. DUL-BiP showed a lower early signal in the technical series, followed by a modest increase on day 6 and a subsequent decline during the remaining cultivation period.

Cleaved ATF6 showed a transient increase in both cultures, with the maximum technical signal occurring later in DUL-BiP than in DUL-Neo, around day 8 rather than day 6. By day 16, cleaved ATF6 was barely quantifiable in either population, precluding reliable calculation of the cleaved-ATF6/ATF6 ratio.

Overall, the semi-quantitative immunoblot data show that fed-batch cultivation was accompanied by temporal changes in cleaved ATF6, eIF2α phosphorylation, and CHOP abundance. Stable constitutive BiP expression was associated with differences in some temporal signal profiles but did not prevent the culture-associated changes in the measured ER-stress-related proteins. Because the analysis was performed using samples from one fed-batch experiment, differences between DUL-Neo and DUL-BiP are interpreted descriptively and do not establish a specific molecular mechanism.

## 4. Discussion

Stable constitutive expression of Chinese hamster BiP was associated with an approximately 40% higher final GLP-1–Fc titer and approximately two additional days of extracellular product accumulation in the single constant-temperature fed-batch experiment. The two populations showed similar viable cell density, viability, and culture duration, indicating that the observed titer difference was not explained by increased biomass or prolonged survival. Because the constant-temperature comparison was performed once biologically, the magnitude of the effect is reported for this experiment rather than treated as an estimate of a population-wide effect size.

The absence of a clear survival phenotype may relate to the properties of the parental host. GLP-1–Fc producer cells were derived from the apoptosis-resistant CHO 4BGD line, which lacks functional BAX and BAK1 and overexpresses Bcl-2 and Beclin-1. This background already limits mitochondrial apoptosis and may leave less scope for the additional cytoprotective effects reported for GRP78-engineered CHO cells under serum deprivation or oxidative stress [12]. In the present system, the production phenotype may therefore be more closely related to ER protein handling than to an extension of cell viability, although the responsible process was not directly measured.

Although the titer curves began to separate before the terminal culture phase, the feature that most clearly distinguished their later kinetics was continued GLP-1–Fc accumulation in DUL-BiP after DUL-Neo approached a plateau. We therefore interpret the phenotype as prolonged late-stage accumulation rather than as an effect arising exclusively in the terminal culture phase. KDELR1 engineering likewise increased IgG1-specific productivity without changing growth rate, with the strongest effect during the deceleration phase [14]. Although KDELR1 influences the retrieval of several ER-resident proteins, both observations are compatible with secretory-pathway constraints becoming increasingly relevant as fed-batch cultures age.

A late-phase contribution is biologically plausible because nutrient availability, energy metabolism, redox balance, metabolite accumulation, and the burden of protein quality control all change during prolonged fed-batch cultivation. Such changes can reduce the functional reserve of the secretory pathway even when cell viability remains relatively high.

In DUL-Neo cells, total BiP increased strongly after day 12, around the period in which extracellular product accumulation was slowing. Because DUL-Neo contains no introduced *Hspa5* cassette, this rise represents induction of endogenous BiP and is consistent with an adaptive response to increasing ER demand. It should not, however, be taken as direct proof that BiP itself was the sole or primary limiting factor. High secretory-protein synthesis and post-translational processing bottlenecks have long been associated with induction of GRP78 and GRP94 in CHO cells [15].

The introduced p1.2-Neo-BiP cassette provides a constitutive *Hspa5* expression input from the EEF1A1 regulatory unit. In DUL-BiP, total BiP showed a comparatively stable temporal profile and did not display the pronounced late increase observed in DUL-Neo. The present immunoblots cannot distinguish transgene-derived from endogenous BiP within DUL-BiP, and the absence of a late rise therefore should not be interpreted as proof that endogenous UPR-driven *Hspa5* induction was suppressed. Rather, the data indicate that introducing the constitutive cassette altered the temporal pattern of total BiP abundance during culture.

BiP function also depends on more than total protein abundance. Client-free BiP can enter reversible oligomeric states or undergo FICD-dependent AMPylation and can be returned to the active client-binding pool as unfolded-protein load changes [9,27,28]. BiP can also participate in ATP-dependent disaggregation of proteins accumulated in the ER under stress [29]. Thus, a large persistent increase in total BiP is not a prerequisite for a biologically relevant change in chaperone availability. In the present study, this provides a plausible framework, not a demonstrated mechanism, for understanding why a constitutive *Hspa5* expression input could influence production kinetics without producing a large sustained fold difference in total BiP.

The UPR-associated protein profiles argue against a simple model in which constitutive BiP expression abolishes ER stress. Cleaved ATF6, eIF2α phosphorylation, and CHOP all changed over cultivation in both populations. Extracellular product therefore continued to accumulate in DUL-BiP while UPR-associated signals were still detectable. Given the single biological sample series, the differences in timing between populations are descriptive and should not be interpreted as proof of pathway-specific regulation by BiP.

This distinction is important because UPR activation is not inherently incompatible with high recombinant-protein productivity. Adaptive ATF6- and IRE1-associated programs can expand folding, quality-control, membrane, and trafficking capacity, whereas prolonged PERK–eIF2α signaling and CHOP induction are associated with translational restriction and loss of productive function. The relevant engineering objective may therefore be preservation of an adaptive ER state rather than complete suppression of UPR signaling.

Pharmacological induction of an ER-chaperone response produces a related but broader phenotype. BiP inducer X increased BiP and other ER chaperones, reduced CHOP and cleaved caspase-3, prolonged stressed CHO cultures, and increased monoclonal antibody accumulation [13]. Because BiP inducer X affects several components of the ER folding system, these effects cannot be assigned specifically to BiP. Our data provide a narrower observation: in the GLP-1–Fc producer examined here, stable constitutive expression of host-derived BiP was associated with prolonged product accumulation without increased growth or complete suppression of the measured UPR-associated signals.

Previous studies show that the outcome of BiP engineering is strongly product-dependent. GRP78 overexpression reduced secretion of von Willebrand factor and blocked secretion of mutant factor VIII, whereas a protein that did not form a stable complex with GRP78 was unaffected [7]. BiP overexpression also reduced IgG-specific productivity in a CHO producer with an imbalance between heavy- and light-chain synthesis [16], where additional BiP reinforced retention of heavy chains that could not complete assembly because the light chain was limiting [17]. These observations are not merely confounding precedents; they define an established biological constraint on the generality of BiP engineering.

GLP-1–Fc differs from these unfavorable examples because the product consists of two identical peptide–Fc chains and does not require coordinated synthesis of separate heavy- and light-chain species. Its maturation still depends on ER translocation, Fc-domain folding, disulfide-bond formation, N-glycosylation, and dimerization. Studies of other Fc-fusion proteins have linked intracellular clipping and aggregation to delayed or incomplete folding, and reduced translation rate or lower cultivation temperature can decrease these defects [18,19,20]. Even peptide–Fc constructs show product-dependent secretion kinetics and glycan heterogeneity in CHO cells [30]. The homodimeric architecture therefore removes one well-defined IgG assembly constraint without making the product independent of ER folding and quality control.

Intracellular GLP-1–Fc did not progressively accumulate in DUL-BiP, and calnexin showed substantially smaller temporal changes than BiP. These observations argue against a simple model of progressively increasing intracellular product retention, but they do not identify the step responsible for the higher extracellular accumulation. A steady-state intracellular immunoblot cannot distinguish altered folding efficiency, secretion rate, proteolytic processing, or intracellular degradation.

Taken together, the production kinetics are compatible with a working model in which the constitutive *Hspa5* cassette changes the timing of BiP supply relative to rising ER demand and thereby helps preserve productive output during the later culture phase. This model is intentionally narrower than a claim that BiP directly increases secretion or folding efficiency. The higher final titer was observed together with prolonged net extracellular product accumulation, while direct secretion rate, folding efficiency, intracellular degradation, and BiP–product interactions were not measured.

A complete Hspa5 knockout would not provide a biologically symmetric counterpart to the present BiP-supplementation strategy. BiP is a central ER chaperone and UPR regulator, and genetic evidence in mammalian systems indicates that substantial loss of BiP can produce primary defects in proliferation, ER homeostasis, and survival. Homozygous Grp78 loss causes peri-implantation lethality with impaired proliferation and apoptosis in the mouse inner cell mass [31], while siRNA-mediated reduction in GRP78 in cultured HeLa cells activates the UPR and promotes apoptosis [32]. These findings do not prove that a stable Hspa5-null CHO population cannot be generated, but they show why complete knockout could confound a production phenotype with a fundamental proteostasis defect. A partial or inducible reduction would be a more interpretable complementary strategy, but it addresses a separate experimental question and was not undertaken in the present study.

The present study examined one GLP-1–Fc-producing parental clone and one independently selected polyclonal population for each transfected construct. Accordingly, the results establish an association within this defined producer system but do not demonstrate clone-independent or population-independent reproducibility, and population-specific effects of random integration or selection cannot be excluded. The two independent temperature-shift fed-batch experiments reproduced the direction of the final-titer difference within these same populations, whereas the detailed constant-temperature phenotype and all immunoblot analyses were obtained from one fed-batch experiment. The absolute titers achieved here were below those typical of optimized industrial monoclonal-antibody processes. However, the parental DUL73 clone previously reached a GLP-1–Fc titer of 1.05 g/L in an optimized 18-day fed-batch process [22], indicating that the present values do not represent its maximal production capacity. The experiments were designed to compare matched populations under identical laboratory fed-batch conditions rather than to optimize process yield. Testing independently generated BiP-engineered populations and additional recombinant products would address the generality and process robustness of the phenotype but is beyond the scope of the present study.

## 5. Conclusions

Stable constitutive expression of the host-derived Chinese hamster Hspa5 coding sequence was associated with prolonged late-stage accumulation of GLP-1–Fc in the producer population examined. In the single constant-temperature fed-batch experiment, DUL-BiP reached an approximately 40% higher final titer without corresponding increases in viable cell density, viability, or culture duration, and extracellular product accumulation continued for approximately two additional days after DUL-Neo had slowed. Two independent temperature-shift experiments showed the same direction of the final-titer difference, although the increases were smaller and accompanied by reduced peak viable cell density. Semi-quantitative immunoblotting of the constant-temperature sample series showed a comparatively stable total BiP trajectory in DUL-BiP and a pronounced late increase in DUL-Neo, together with temporal changes in ATF6 forms, eIF2α phosphorylation, and CHOP in both populations. These immunoblot data are descriptive and do not establish a specific molecular mechanism. The findings are compatible with a working model in which the timing of BiP supply relative to ER demand influences the maintenance of productive output, but direct secretion rate, folding efficiency, intracellular degradation, and BiP–product interactions were not measured. The present results therefore support further evaluation of BiP engineering in independently generated producer populations and additional recombinant products rather than a universal conclusion about BiP as a CHO productivity target.

## Data Availability

The numerical source data supporting the cell-culture and densitometric analyses, together with uncropped annotated Western blot images, are provided in the Supplementary Materials.

## Supplementary Materials

The following supporting information can be downloaded at: https://www.mdpi.com/article/10.3390/bioengineering13091001/s1; Supplementary File S1: Uncropped Western blot images, image overlays with molecular-weight markers, and an index file with image descriptions. Dataset S1: Numerical source data and calculations supporting Figure 2, Figure 3, Figure 4 and Figure 5.

## Abbreviations

The following abbreviations are used in this manuscript:

ATF6	Activating transcription factor 6
BiP	Binding immunoglobulin protein
CHO	Chinese hamster ovary
CHOP	C/EBP homologous protein
CNX	Calnexin
eIF2α	Eukaryotic translation initiation factor 2α
ER	Endoplasmic reticulum
GLP-1–Fc	Glucagon-like peptide-1–Fc fusion protein
IVCD	Integral viable cell density
MTX	Methotrexate
qP	Cell-specific productivity
UPR	Unfolded protein response
VCD	Viable cell density

## References

[1] Bai Y., Mercade I.D., Elgendy R., Lambiase G., Peak-Chew S., Franco C., Wingett S.W., Stevens T.J., Grassi L., Hitchcock N. (2025). Identification of Cellular Signatures Associated with Chinese Hamster Ovary Cell Adaptation for Secretion of Antibodies. Comput. Struct. Biotechnol. J..

[2] Rives D., Peak C., Blenner M.A. (2024). RNASeq Highlights ATF6 Pathway Regulators for CHO Cell Engineering with Different Impacts of ATF6β and WFS1 Knockdown on Fed-Batch Production of IgG1. Sci. Rep..

[3] Schulz A., Munro T., Puklowski A., Slack E., Tolstrup A.B., Otte K. (2026). Systematic Review and Data-Driven Insights into CHO Cell Engineering for Next-Generation Antibody Production. mAbs.

[4] Carlage T., Hincapie M., Zang L., Lyubarskaya Y., Madden H., Mhatre R., Hancock W.S. (2009). Proteomic Profiling of a High-Producing Chinese Hamster Ovary Cell Culture. Anal. Chem..

[5] Orlova N.A., Kovnir S.V., Gabibov A.G., Vorobiev I.I. (2017). Stable High-Level Expression of Factor VIII in Chinese Hamster Ovary Cells in Improved Elongation Factor-1 Alpha-Based System. BMC Biotechnol..

[6] Nissom P.M., Sanny A., Kok Y.J., Hiang Y.T., Chuah S.H., Shing T.K., Lee Y.Y., Wong K.T.K., Hu W.-S., Yap M.G.S. (2006). Transcriptome and Proteome Profiling to Understanding the Biology of High Productivity CHO Cells. Mol. Biotechnol..

[7] Dorner A.J., Wasley L.C., Kaufman R.J. (1992). Overexpression of GRP78 Mitigates Stress Induction of Glucose-Regulated Proteins and Blocks Secretion of Selective Proteins in Chinese Hamster Ovary Cells. EMBO J..

[8] Wang X.Z., Lawson B., Brewer J.W., Zinszner H., Sanjay A., Mi L.J., Boorstein R., Kreibich G., Hendershot L.M., Ron D. (1996). Signals from the Stressed Endoplasmic Reticulum Induce C/EBP-Homologous Protein (CHOP/GADD153). Mol. Cell. Biol..

[9] Pobre K.F.R., Poet G.J., Hendershot L.M. (2019). The Endoplasmic Reticulum (ER) Chaperone BiP Is a Master Regulator of ER Functions: Getting by with a Little Help from ERdj Friends. J. Biol. Chem..

[10] Wiseman R.L., Mesgarzadeh J.S., Hendershot L.M. (2022). Reshaping Endoplasmic Reticulum Quality Control through the Unfolded Protein Response. Mol. Cell.

[11] Obaseki I., Ndolo C.C., Adedeji A.A., Popoola H.O., Kravats A.N. (2025). The Structural and Functional Dynamics of BiP and Grp94: Opportunities for Therapeutic Discovery. Trends Pharmacol. Sci..

[12] Jiang Q., Sun Y., Guo Z., Fu M., Wang Q., Zhu H., Lei P., Shen G. (2017). Overexpression of GRP78 Enhances Survival of CHO Cells in Response to Serum Deprivation and Oxidative Stress. Eng. Life Sci..

[13] Ha T.K., Hansen A.H., Kildegaard H.F., Lee G.M. (2019). BiP Inducer X: An ER Stress Inhibitor for Enhancing Recombinant Antibody Production in CHO Cell Culture. Biotechnol. J..

[14] Samy A., Kaneyoshi K., Omasa T. (2020). Improvement of Intracellular Traffic System by Overexpression of KDEL Receptor 1 in Antibody-Producing CHO Cells. Biotechnol. J..

[15] Dorner A.J., Wasley L.C., Kaufman R.J. (1989). Increased Synthesis of Secreted Proteins Induces Expression of Glucose-Regulated Proteins in Butyrate-Treated Chinese Hamster Ovary Cells. J. Biol. Chem..

[16] Borth N., Mattanovich D., Kunert R., Katinger H. (2005). Effect of Increased Expression of Protein Disulfide Isomerase and Heavy Chain Binding Protein on Antibody Secretion in a Recombinant CHO Cell Line. Biotechnol. Prog..

[17] Ho S.C.L., Koh E.Y.C., van Beers M., Mueller M., Wan C., Teo G., Song Z., Tong Y.W., Yang Y. (2013). Control of IgG LC:HC Ratio in Stably Transfected CHO Cells and Study of the Impact on Expression, Aggregation, Glycosylation and Conformational Stability. J. Biotechnol..

[18] Chakrabarti S., Barrow C.J., Kanwar R.K., Ramana V., Kanwar J.R. (2016). Studies to Prevent Degradation of Recombinant Fc-Fusion Protein Expressed in Mammalian Cell Line and Protein Characterization. Int. J. Mol. Sci..

[19] Henry M., Gallagher C., Kelly R.M., Frye C.C., Osborne M.D., Brady C.P., Barron N., Clynes M., Meleady P. (2018). Clonal Variation in Productivity and Proteolytic Clipping of an Fc-Fusion Protein in CHO Cells: Proteomic Analysis Suggests a Role for Defective Protein Folding and the UPR. J. Biotechnol..

[20] Knight T.J., Povey J.F., Vito D., Mohindra A., Jaques C.M., Smales C.M. (2022). Manipulation of mRNA Translation Elongation Influences the Fragmentation of a Biotherapeutic Fc-Fusion Protein Produced in CHO Cells. Biotechnol. Bioeng..

[21] Glaesner W., Vick A.M., Millican R., Ellis B., Tschang S.-H., Tian Y., Bokvist K., Brenner M., Koester A., Porksen N. (2010). Engineering and Characterization of the Long-Acting Glucagon-Like Peptide-1 Analogue LY2189265, an Fc Fusion Protein. Diabetes Metab. Res. Rev..

[22] Shaifutdinov R.R., Sinegubova M.V., Vorobiev I.I., Prokhorova P.E., Podkorytov A.B., Orlova N.A. (2025). Production of a Dulaglutide Analogue by Apoptosis-Resistant Chinese Hamster Ovary Cells in a 3-Week Fed-Batch Process. Pharmaceuticals.

[23] Orlova N.A., Sinegubova M.V., Kolesov D.E., Khodak Y.A., Tatarskiy V.V., Vorobiev I.I. (2025). Genomic and Phenotypic Characterization of CHO 4BGD Cells with Quad Knockout and Overexpression of Two Housekeeping Genes That Allow for Metabolic Selection and Extended Fed-Batch Culturing. Cells.

[24] Sinegubova M.V., Orlova N.A., Vorobiev I.I. (2023). Promoter from Chinese Hamster Elongation Factor-1α Gene and Epstein–Barr Virus Terminal Repeats Concatemer Fragment Maintain Stable High-Level Expression of Recombinant Proteins. PeerJ.

[25] Orlova N.A., Kovnir S.V., Hodak J.A., Vorobiev I.I., Gabibov A.G., Skryabin K.G. (2014). Improved Elongation Factor-1 Alpha-Based Vectors for Stable High-Level Expression of Heterologous Proteins in Chinese Hamster Ovary Cells. BMC Biotechnol..

[26] Shaifutdinov R.R., Sinegubova M.V., Vorobiev I.I., Orlova N.A. (2026). Plasmids for Expression of Recombinant Human Glucagon-Like Peptide-1 Receptor Agonist Fused to Human Immunoglobulin Crystallizable Fragment (Dulaglutide), Monoclonal Mammalian Cell Line Producing Dulaglutide, Method for Producing Dulaglutide.

[27] Preissler S., Chambers J.E., Crespillo-Casado A., Avezov E., Miranda E., Perez J., Hendershot L.M., Harding H.P., Ron D. (2015). Physiological Modulation of BiP Activity by Trans-Protomer Engagement of the Interdomain Linker. eLife.

[28] Preissler S., Rato C., Chen R., Antrobus R., Ding S., Fearnley I.M., Ron D. (2015). AMPylation Matches BiP Activity to Client Protein Load in the Endoplasmic Reticulum. eLife.

[29] Pinho Melo E., Konno T., Farace I., Awadelkareem M.A., Skov L.R., Teodoro F., Sancho T.P., Paton A.W., Paton J.C., Fares M. (2022). Stress-Induced Protein Disaggregation in the Endoplasmic Reticulum Catalysed by BiP. Nat. Commun..

[30] Carlson K.R., Pomerantz S.C., Li J., Vafa O., Naso M., Strohl W., Mains R.E., Eipper B.A. (2015). Secretion of Fc-Amidated Peptide Fusion Proteins by Chinese Hamster Ovary Cells. BMC Biotechnol..

[31] Luo S., Mao C., Lee B., Lee A.S. (2006). GRP78/BiP Is Required for Cell Proliferation and Protecting the Inner Cell Mass from Apoptosis during Early Mouse Embryonic Development. Mol. Cell. Biol..

[32] Suzuki T., Lu J., Zahed M., Kita K., Suzuki N. (2007). Reduction of GRP78 Expression with siRNA Activates Unfolded Protein Response Leading to Apoptosis in HeLa Cells. Arch. Biochem. Biophys..

